# The Identification of Small RNAs Differentially Expressed in Apple Buds Reveals a Potential Role of the Mir159-MYB Regulatory Module during Dormancy

**DOI:** 10.3390/plants10122665

**Published:** 2021-12-03

**Authors:** Julio Garighan, Etienne Dvorak, Joan Estevan, Karine Loridon, Bruno Huettel, Gautier Sarah, Isabelle Farrera, Julie Leclercq, Priscila Grynberg, Roberto Coiti Togawa, Marcos Mota do Carmo Costa, Evelyne Costes, Fernando Andrés

**Affiliations:** 1 UMR AGAP Institute, Institut Agro, CIRAD, INRAE, University of Montpellier, F-34398 Montpellier, France; julio.garighan@inrae.fr (J.G.); etienne.dvorak@inrae.fr (E.D.); joan.estevan@inrae.fr (J.E.); karine.loridon@inrae.fr (K.L.); gautier.sarah@inrae.fr (G.S.); isabelle.farrera@supagro.fr (I.F.); julie.leclercq@cirad.fr (J.L.); evelyne.costes@inrae.fr (E.C.); 2 Genome Centre, Max Planck Institute for Plant Breeding Research, D-50829 Cologne, Germany; huettel@mpipz.mpg.de; 3 UMR AGAP Institute, CIRAD, F-34398 Montpellier, France; 4 Bioinformatica Laboratory, Embrapa Recursos Genéticos e Biotecnologia—Cenargen, Brasilia 02372, Brazil; priscila.grynberg@embrapa.br (P.G.); roberto.togawa@embrapa.br (R.C.T.); marcos.costa@embrapa.br (M.M.d.C.C.)

**Keywords:** dormancy, apple tree, small RNAs, miR159

## Abstract

Winter dormancy is an adaptative mechanism that temperate and boreal trees have developed to protect their meristems against low temperatures. In apple trees (*Malus domestica*), cold temperatures induce bud dormancy at the end of summer/beginning of the fall. Apple buds stay dormant during winter until they are exposed to a period of cold, after which they can resume growth (budbreak) and initiate flowering in response to warmer temperatures in spring. It is well-known that small RNAs modulate temperature responses in many plant species, but however, how small RNAs are involved in genetic networks of temperature-mediated dormancy control in fruit tree species remains unclear. Here, we have made use of a recently developed ARGONAUTE (AGO)-purification technique to isolate small RNAs from apple buds. A small RNA-seq experiment resulted in the identification of 17 micro RNAs (miRNAs) that change their pattern of expression in apple buds during dormancy. Furthermore, the functional analysis of their predicted target genes suggests a main role of the 17 miRNAs in phenylpropanoid biosynthesis, gene regulation, plant development and growth, and response to stimulus. Finally, we studied the conservation of the *Arabidopsis thaliana* regulatory miR159-MYB module in apple in the context of the plant hormone abscisic acid homeostasis.

## 1. Introduction

Temperate trees spread over geographical regions presenting wide seasonal environmental fluctuations all over the year. In order to optimize their reproductive success, they adjust their growth and flowering cycles to these recurrent conditions. This is possible thanks to environment sensing mechanisms and signaling pathways that reprogram their meristems in response to changes of photoperiod and temperature. In winter, short photoperiods and low temperatures induce growth cessation, the formation of winter buds to protect the meristematic tissues, and a phase of meristem growth inhibition named endodormancy (or winter dormancy) [1]. Endodormant buds regain their competence to grow after exposure to a certain period of low temperatures that is known as chilling requirement (CR). Once CR is fulfilled, the shoot apical meristem (SAM) undergoes an ecodormant phase. During ecodormancy, warm temperatures typical of springtime lead to growth resumption, budbreak and flowering [2,3].

The Rosaceae is the third most economically important plant family in temperate regions and responsible for the major part of the total worldwide-consumed fruits [4]. Iconic Rosaceous species such as apple (*Malus domestica*), peach (*Prunus persica*) and sweet cherry (*Prunus avium*) are characterized by high CRs. This feature limits the range of latitudes at which they can be productively grown and makes them highly susceptible to global warming. However, despite the importance of a well-adjusted dormancy cycle for flowering timing and fruit production, the understanding on how this process is controlled in Rosaceous species and other significant crops is still in its infancy. A group of genes encoding MADS transcription factors (TF) are believed to be major regulators of the dormancy cycle in many Rosaceous fruit trees [3,5,6,7]. Some of these MADS TF are named as *DORMANCY-ASSOCIATED MADS-BOX* (*DAM*) because of their genetic association with the non-dormant phenotype of the *evg* mutant of peach [6]. *DAM* genes belong to the MIKC^C^ type of MADS TF genes and are similar to *SHORT VEGETATIVE PHASE* (*SVP*) from *Arabidopsis thaliana* [8]. They associate in transcriptional complexes that act in an intricate gene regulatory network (GRN) that control the transition between dormancy phases [3,5,9]. This GRN seems to be regulated, at least in part, by the hormonal and environment-mediated transcriptional modulation of its key components [5,7,10]. For example, the plant hormone abscisic acid (ABA) is a positive regulator of dormancy induction in several tree species [11,12,13]. Although some recent studies have shed light on mechanisms of transcriptional regulation of bud dormancy in trees, how it is controlled at the post-transcriptional level remains unclear.

Most of the plant small RNAs consist in 21 to 24-nucleotide RNA molecules produced by DICER-LIKE (DCL) proteins [14]. Small RNAs are loaded onto ARGONAUTE (AGO)-like proteins to induce mRNA cleavage, translational repression, chromatin compaction or DNA elimination [15,16,17]. They are widely involved in the regulation of plant development, reproduction, responses to the environment, biotic and abiotic stress and genome reprogramming [18,19]. The micro RNAs (miRNAs) are a class of small RNAs that in plants mediate post-transcriptional gene silencing (PTGS) by transcript cleavage or translational repression [18]. In *A. thaliana*, the miR156 and miR172 are key regulators of flowering through the post-transcriptional regulation of *SQUAMOSA PROMOTER-BINDING PROTEIN-LIKE* (*SPL*) and *APETALA2*-like (*AP2*-like) genes, respectively [20,21,22]. Interestingly, miR156 and miR172 were found to be also involved in seed dormancy in lettuce (*Lactuca sativa*) and *A. thaliana* [23]. However, reports describing the role of small RNAs in bud dormancy are scarce. Only a few recent studies using genome-wide small RNA sequencing (small RNA-seq) identified potential miRNAs that regulate dormancy in trees [24,25,26,27]. In tree peony, several miRNAs were found to be differentially accumulated in buds between endodormancy and ecodormancy after a period of chilling. Among the identified miRNAs, five of them (miR156k, miR159a, miR167a, miR169a and miR172a) showed an inverse expression pattern as compared to their target genes [27], suggesting their role during endodormancy to ecodormancy transition in tree peony. Another recent study reported the existence of miRNA-mediated regulating the photoperiod-dependent dormancy induction in Vitis species [28]. Most of the studies concerning Rosaceous species have been done on pear and peach [24,26,29,30]. More than hundred miRNAs were expressed in buds during dormancy of Japanese pear (*Pyrus pyrifolia* ‘Kosui’) [26]. In white pear (*Pyrus pyrifolia*), it was found that miRNA6390 targeted several *DAM* genes as a part of a potential mechanism of endodormancy release [24]. A *Prunus*-specific miRNA (miR6285) was also found to be differentially expressed between endodormancy and ecodormancy in peach. MiR6285 targets an asparagine-rich protein (NRP) that is involved in the regulation of ABA signaling in *A. thaliana* [29].

Here, we have made use of a recently developed AGO-purification technique [31] to isolate small RNAs from apple buds. Combining small RNA-seq and public RNA-seq data allowed us to identify miRNA and their target genes potentially involved in the control of dormancy cycle of fruit trees. Furthermore, we studied the conservation between *A. thaliana* and apple of a regulatory miRNA/target gene module in the context of the ABA homeostasis.

## 2. Results

### 2.1. Different Types of Known and Unknown Small RNAs Are Expressed in Apple Buds during the Dormancy Cycle

**Figure 1:**
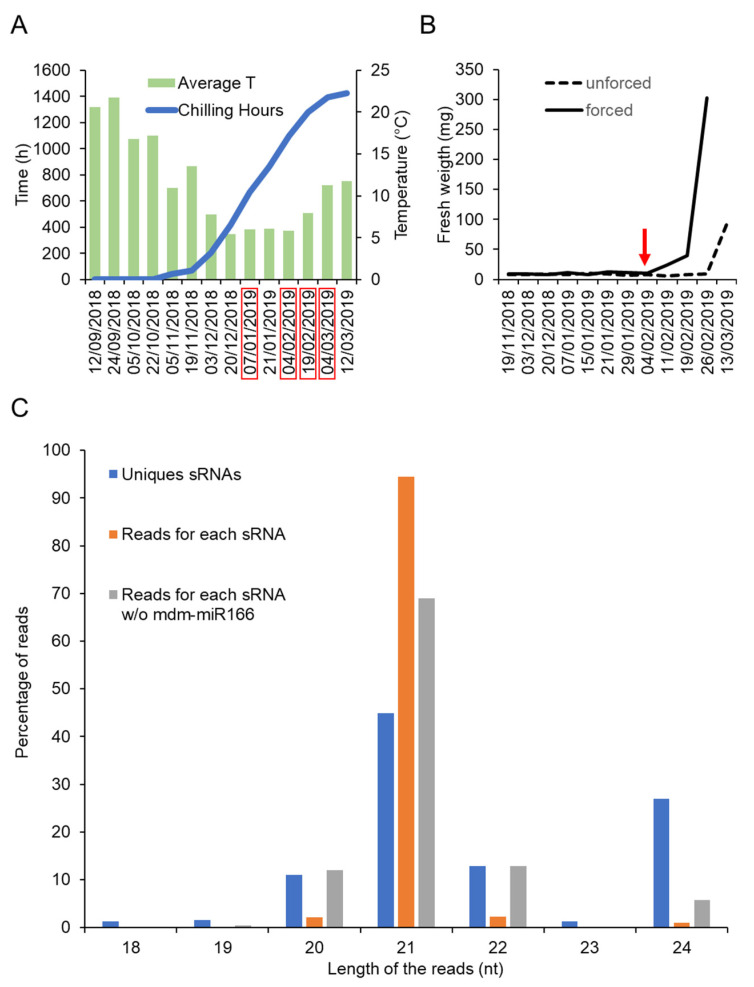
Identification of small RNAs expressed during dormancy of apple buds. (**A**) Average temperature and chilling hours accumulation during the experiment done between 2018 and 2019. (**B**) Result of the Tabuenca test showing the around which endodormancy is released (red arrow) (**C**) Percentage of reads corresponding to the number of distinct types of small RNAs (Unique sRNAs), to the nucleotide lengths distribution (Reads for each sRNA) with or without mdm-miR166 (Reads for each sRNA w/o mdm-mR166).

In order to identify small RNAs expressed during dormancy cycle in apple, bud samples were collected at different dates covering the transition between endodormancy to ecodormancy. Four dates were selected according to the physiological stage of the buds (Table 1 and Figure 1A,B). The physiological stage of the buds was defined using a forcing test (Tabuenca test [32,33]) that allowed us to distinguish between endodormant and ecodormant buds (Figure 1B). Based on the Tabuenca test that we periodically performed between November 2018 and March 2019, we determined that the transition from endodormancy to ecodormancy occurred around 04/02/2019 (Stage 2, Table 1). Before this date, buds remained fully endodormant (Stage 1), whereas after this date, buds became ecodormant (Stages 3 and 4), i.e., competent to resume growth upon exposure to warm T° (Figure 1A and Table 1).

To isolate small RNAs from these samples, we have made use of a recently developed method called “TraPR”, which purification protocol is based on AGO’s ability to associate with small RNA. The sequencing results after small RNA isolation using this method are shown in Table 1. A total of 372 different small RNAs were detected across samples, among which, 361 corresponded to biologically relevant small RNAs (i.e., length between 20 to 24 nucleotides) (Table S1). The size distribution shown in Figure 1C indicates a predominant abundance of 21-nucleotides small RNAs. Notably, around one third (28%) of the detected unique small RNAs corresponded to 24-nucleotides small RNAs, which could mediate transcriptional silencing of transposons and pericentromeric repeats via the RdDM pathway [18]. A total of 233 small RNAs were annotated in public databases as known miRNAs, whereas the other 128 were annotated as unknown small RNAs (Table S1). Particularly abundant were the miRNAs belonging to the miR166, miR159, miR482, miR1511, miR319 and miR171 families. Remarkably, only the miR166 accounted for around 80% of the total recovered reads (Table S1).

### 2.2. Several miRNAs Are Differentially Expressed between Endodormancy and Ecodormancy in Apple

**Figure 2:**
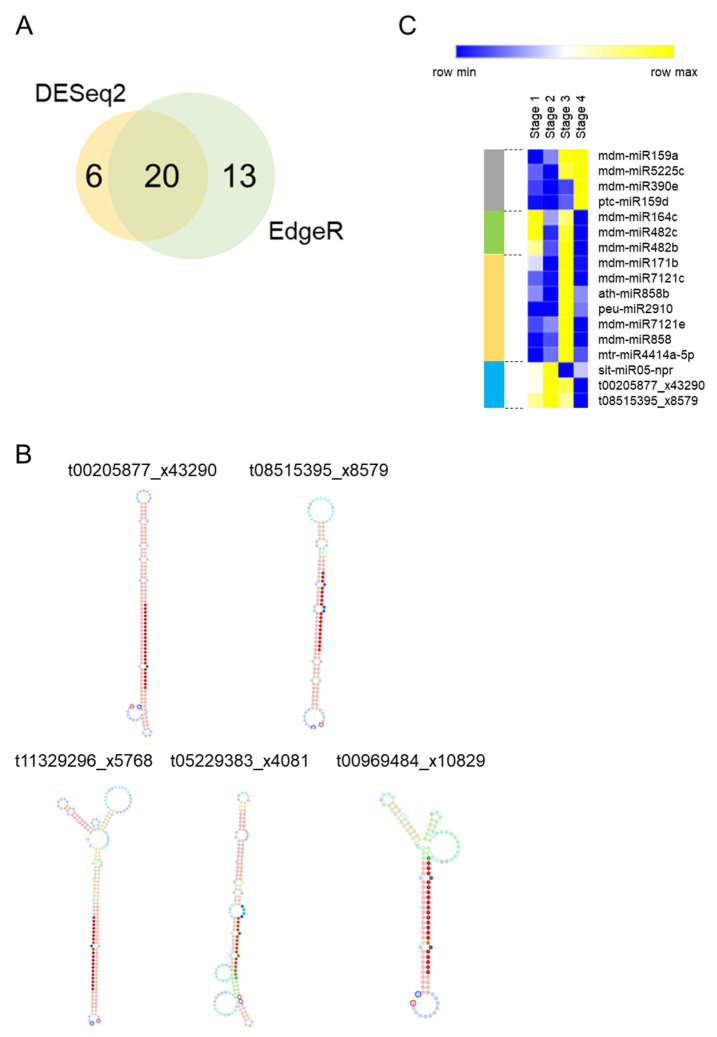
Several small RNAs are differentially expressed between endodormancy to ecodormancy in apple buds. (**A**) Venn Diagram comparing the results between the EdgerR and Deseq2 analyses. (**B**) Stem-loop structure prediction of unknown DE-small RNAs. (**C**) Heat map Expression profile of the 17 DE-miRNAs.

We tested for small RNA differential expression using two R packages (DESeq2 and Edge2). An analysis performed with DESeq2 resulted in the detection of 26 differentially expressed small RNAs (DE-small RNAs), while EdgeR found 33 DE-small RNAs (Supplementary Table S2; *p*-value ≤ 0.05). A group of 20 DE-small RNAs was common between the two analyses (Figure 2A). This group was considered as a high confidence list of DE-small RNAs. The high confident list contained five small RNAs (t11329296_x5768, t05229383_x4081, t08515395_x8579, t00205877_x43290 and t00969484_x10829) that were not included in any public miRNA database. Therefore, we performed a further analysis to evaluate their stem-loop structures. In silico RNA folding analyses predicted that only t08515395_x8579 and t00205877_x43290 have an optimal stem-loop structure compatible with biologically functional miRNAs [34] (Figure 2B). Therefore, the other three small RNAs (t11329296_x5768, t05229383_x4081 and t00969484_x10829) were discarded for downstream studies. Distinct patterns of expression were observed within the 17 DE-miRNAs of the high confidence list (Table 2 and Figure 2C). Four miRNAs were upregulated at Stage 4 (grey bar in Figure 2C). Two of them, mdm-miR159a and mdm-miR5225c increased their expression at the transition between endodormancy to ecodormancy. Similarly, a group of seven miRNAs (orange bar) were upregulated at Stage 3 but their expression levels dropped at Stage 4. Three miRNAs displayed reduced levels of expression at Stage 4 compared to Stages 1 and 2 (blue bar) and other three ones showed two peaks of expression at Stages 1 and 3 (green bar).

### 2.3. Predicted Target Genes of DE-miRNAs Are Involved in Key Regulatory Biological Processes

**Figure 3:**
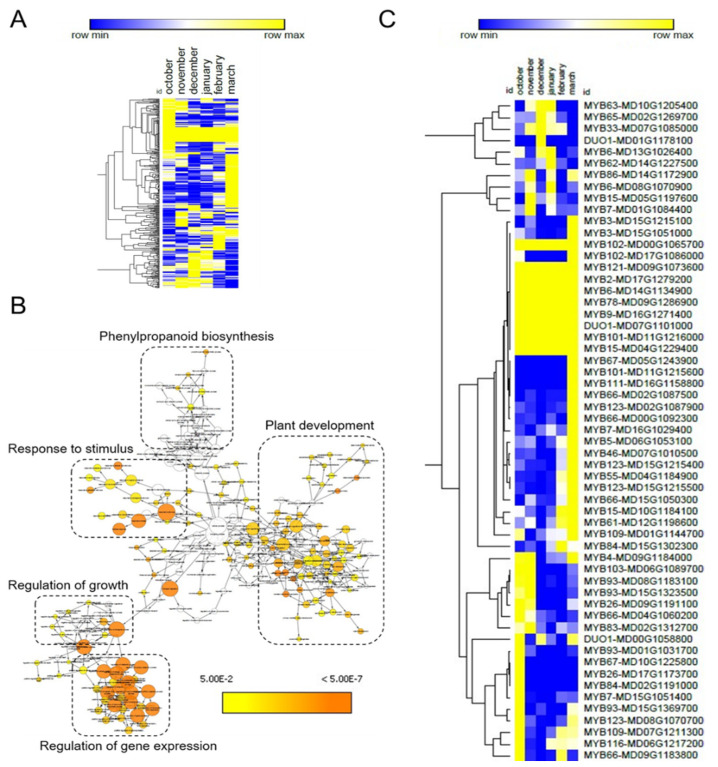
GO enrichment of DE-miRNA targets. (**A**) Heat map of DE-miRNA target genes patterns of expression. (**B**) Schematic map showing the GO enrichment of the DE-miRNA target genes. A high-resolution image is available in the Figure S1. (**C**) Heat map of target genes of DE-small RNA encoding MYB transcription factors. In (**A**,**C**), blue and yellow colours mean upregulation and downregulation, respectively.

We made use of the psRNATarget web server for the identification of target genes of the DE-miRNAs. This analysis resulted in the identification of 420 potential miRNA target genes (Table S3). One quarter of these genes (101 genes) were common targets among the 17 DE-miRNAs. Next, we made use of public RNA-seq data [35] to represent the expression profile of the 319 unique target genes in a heat map. Several target genes displayed changing expression patterns in apple buds collected at distinct dates during winter dormancy (Figure 3A and Figure S1), suggesting their possible role in this biological process. To investigate the biological function of these target genes, we performed a gene ontology (GO) enrichment analysis. This analysis resulted in the classification of the 319 target genes into five main categories related to plant development, regulation of gene transcription, phenylpropanoid biosynthesis, regulation of growth and response to stimulus (Figure 3B and Table S4). Categories related to plant development included genes involved in meristem and flower development, as well as xylem formation and specification of symmetry. Remarkably, genes with roles related to response to hormones, i.e., ABA, jasmonic acid, gibberellin and auxin, were overrepresented in the category response to stimulus. Moreover, the category regulation of growth included genes related to cell development and proliferation among others. Finally, several genes encoding transcription factors were found within the category of regulation of gene expression.

We found that 18% of the genes showing a GO enrichment (57 genes) were annotated as MYB transcription factors. These MYB-encoding genes were targeted by many of the 17 DE-miRNAs, suggesting their role in a post-transcriptional network regulating bud dormancy. Corroborating this idea, several of these *MYB* genes were differentially expressed during the dormancy cycle (Figure 3C).

### 2.4. The ABA Regulatory Module Mir159a-MYB33/MYB65 Is Conserved in Apple

**Figure 4:**
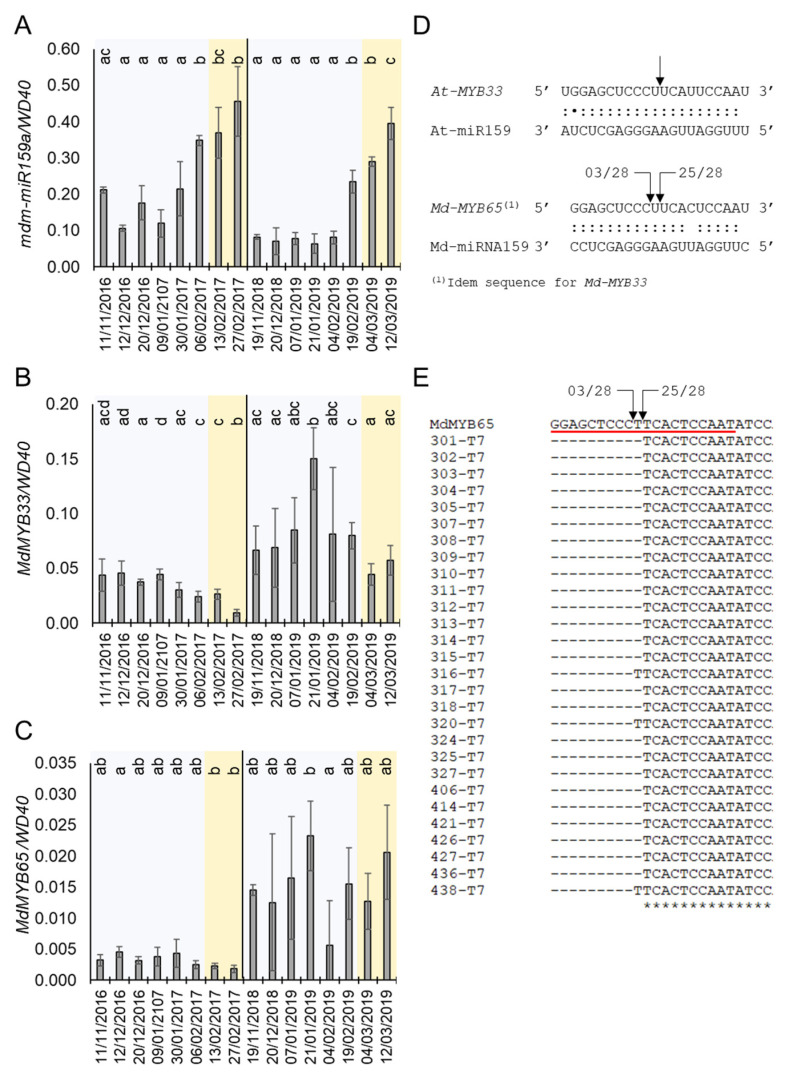
Expression of mdm-miR159and their targets during dormancy and in response to ABA. (**A**) Annual expression profile of pre-mdm-miR159a. (**B**) Annual expression profile of *MdMYB33*. (**C**) Annual expression profile of *MdMYB65*. (**D**) Comparison between *A. thaliana* and apple of target sites of miR159 on *MYBs*. (**E**) Alignment of the miR159 target site (red underline) and the sequences recovered from 28 independent clones resulted from the 5′ RLM-RACE assay. In (**D**,**E**), arrows indicate the 5′ position of the cleaved mRNA fragment identified by RLM-5′ RACE, and the numbers refer to the number of independent clones analyzed. In (**A**–**C**), blue and orange shadows mean endodormancy and ecodormancy phases, respectively. Statistical analysis was done using a *t*-test. Letters shared in common between the dates indicate no significant differences (for *p* ≤ 0.01).

Our GO analysis suggested a role of ABA in apple bud dormancy control mediated by miRNAs. ABA is a well-known hormonal regulator of dormancy in tree species [13,36,37]. Interestingly, one of the identified 17 apple DE-miRNAs is annotated as mdm-miRNA159a, which homolog in *A. thaliana* modulates ABA signalling by targeting genes encoding R2R3 MYB transcription factors [38]. According to our small RNA-seq, mdm-miRNA159a expression stayed low during Stages 1 and 2 (endodormancy) and increased during Stages 3 and 4 (ecodormancy) (Figure 2B). This pattern of expression was confirmed by quantifying the pre-miRNA level of mdm-miRNA159a during the dormancy cycle in apple buds (Figure 4A). The mRNA level of its putative target genes *MdMYB33* and *MdMYB65* was also differentially expressed during the dormancy cycle (Figure 3C and Figure 4B,C). As shown in the Figure 4B, *MdMYB33* mRNA was upregulated during endodormancy (between December and January) and strongly downregulated in the ecodormancy (from February to March), coinciding with the maximum accumulation of mdm-mRNA159a pre-miRNA levels (Figure 4A). Although the expression of *MdMYB65* mRNA was similar to that shown for *MYB33* in the years 2016/2017, this pattern was not reproduced in 2018/2019 (Figure 4C). The downregulation of *MdMYB33* and *MdMYB65* expression could be caused by the activity of mdm-miR159a, since these loci contain a canonical miR159 binding site [38] (Figure 4D). Moreover, the miR159a binding site on *MdMYB33* was empirically demonstrated by degradome sequencing [39].

Furthermore, we determined the precise mdm-miRNA159a cleavage site on the *MdMYB65* gene by 5′ RNA ligase-mediated rapid amplification of cDNA ends (5′ RLM-RACE). Notably, we detected two potential cleavage sites within the miR159a binding sequence on *MdMYB65* (which is identical to the one in *MdMYB33).* The most frequent cleaved mRNA fragment, supported by 25 out of 28 independent clones, coincides with the same nucleotide sequence that was shown for *Arabidopsis* (Figure 4C,D).

**Figure 5:**
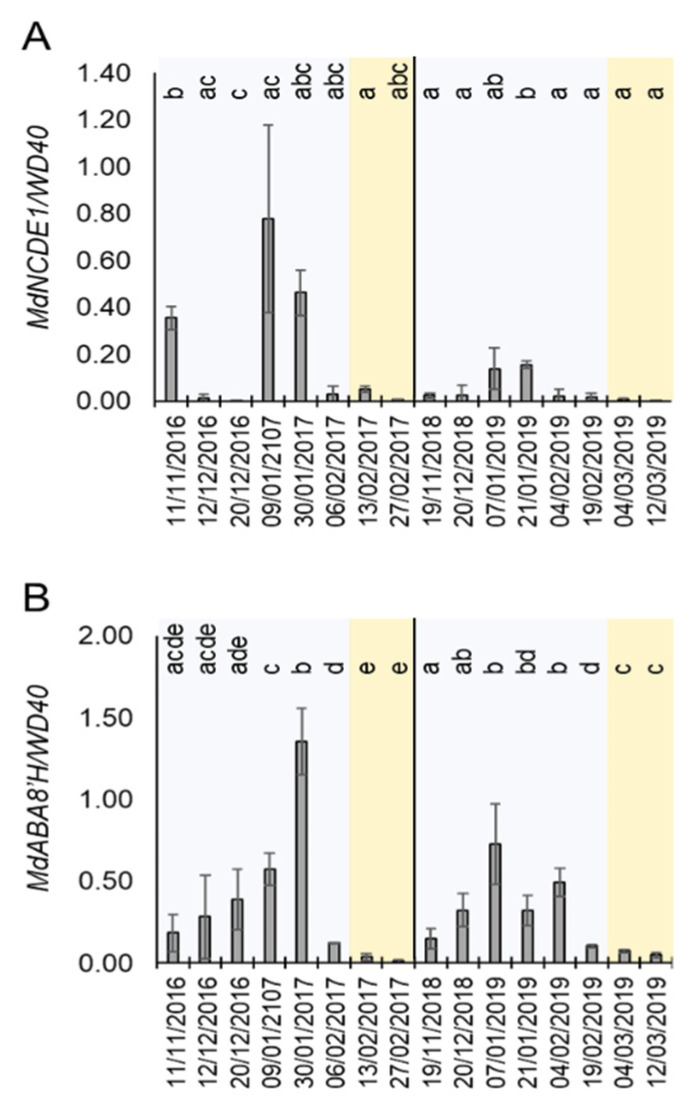
Expression of ABA-responsive genes during dormancy. (**A**) Relative expression of *MdNCED1*. (**B**) Relative expression of *MdABA8′H.* Blue and orange shadows mean endodormancy and ecodormancy phases, respectively. Statistical analysis was done using a *t*-test. Letters shared in common between the dates indicate no significant differences (for *p* ≤ 0.01).

The above-mentioned results suggested a role of the mdm-miR159a and its targets *MdMYB33* and *MdMYB65* during dormancy cycle. In *A. thaliana*, miR159a is induced by ABA to reduce the sensitivity to this hormone in a feedback loop that involves the repression of these *MYB* genes [38]. In order to correlate the expression of miRNA159 and the ABA metabolism during dormancy, we monitored the level of expression of ABA biosynthetic and catabolic genes in buds in the same time-course experiment shown in Figure 4. Remarkably, the expression of *MdNCDE1*, a gene that encodes the ABA biosynthetic enzyme 9-cis-epoxycarotenoid dioxygenase [40], was induced at certain dates during endodormancy (winter period) and highly reduced during ecodormancy. This result suggested an ABA biosynthetic activity during the endodormancy phase. Furthermore, the mRNA level of *MdABA8′H*, which encodes an ABA 8′-hydroxylase enzyme involved in ABA catabolism [40], was clearly upregulated during endodormacy and strongly repressed in ecodormancy (Figure 5B). As *MdABA8′H* mRNA expression is induced by ABA [40], together with the *MdNCED1* expression profile (Figure 5A), this result corroborates previous reports proposing that highest ABA levels are found during endodormancy and negatively correlates with budbreak in different fruit tree species, including apple, sour cherry, sweet cherry, pear and peach [12,37,41,42]. This result suggested that miR159a is induced by ABA during apple bud dormancy.

## 3. Discussion

### 3.1. Seventeen DE-miRNAs Could Be Part of GRNs Controlling Bud Dormancy in Apple

The transition from endodormancy to ecodormancy and the activation of budbreak is believed to be controlled by complex GRNs that integrate endogenous and environmental signals [3,5,43]. TFs are key players in these GRNs, and a timed and coordinated control of their gene expression seems essential to orchestrate dormancy phase transitions [3,5]. This tight regulation is likely to occur not only at the transcriptional level but also post-transcriptionally. MiRNAs are regulatory molecules that exert a large part of their function through the post-transcriptional inhibition of major transcription factors [44]. Furthermore, miRNA can diffuse across tissues and act as long-distance signals [45] making them central players in developmental GRNs, as those regulating dormancy in trees. The sampling of apical dormant buds from a homogeneous shoot type (spurs) may have favoured the detection of miRNAs, as such buds are synchronized in their developmental stages up to outgrowth. Our small-RNAseq approach and data analysis resulted in the identification of 17 DE-miRNAs possibly involved in GRN controlling bud dormancy of apple. Within these 17 miRNAs, some of them were already found DE during bud dormancy in other tree species. This is the case of members of the miRNA families miR390, miR858, miR164 and miR159 that were also DE during dormancy in tree peony [27] and Japanese pear [26]. However, the observed miRNA expression patterns are not always consistent among species, which suggest a partial evolutionary divergence on the mechanisms controlling dormancy across taxa. Supporting this idea, two recently described miRNAs, miR6390 and miR6285, in white pear [24] and peach [29], respectively, were not detected in apple buds during dormancy. Nevertheless, the 17 DE-miRNAs we identified here are likely to participate in dormancy-related GRNs. Confirming this hypothesis will require a further molecular and functional validation of these miRNAs and their target genes.

### 3.2. Potential Roles of Target Genes of DE-miRNAs during Bud Dormancy of Apple

In silico prediction identified more than 300 target genes for the 17 DE-miRNAs. The functional classification of these potential target genes based on GO enrichment, allow us to hypothesize on the role of the DE-miRNAs in apple dormancy. Corroborating the expected role of the DE-miRNAs in dormancy-associated GRNs, a large part of the predicted target genes encoded TFs, and in particular MYB TFs, that were included in the GO term of regulation of gene expression. The DE-miRNAs’ potential targets were also associated with GO terms related to phenylpropanoid biosynthesis, plant development and growth and response to stimulus. Interestingly, phenylpropanoids are secondary metabolites that include flavonoids, monolignols, phenolic acids, stilbenes and coumarin, and have essential roles in plant development. Their function has been already related to bud dormancy in trees. For instance, changes in the expression of genes involved in phenylpropanoid pathway during bud dormancy were reported in apricot (*Prunus armeniaca*) grapevine (*Vitis* species) and pear (*Pyrus pyrifolia*) [46,47,48,49]. Moreover, some flavonoids (e.g., kaempferol and quercetin) have functions as auxin transport and redox scavengers [50,51,52] and could be involved thus in bud dormancy and growth resumption [53,54]. In *A. thaliana*, miR858 silences the expression of several MYB TFs involved in the phenylpropanoid pathway, including MYB11, 12, 111and 123 [55]. We found mdm-miR858 and ath-miR858b among the identified 17 DE-miRNAs and both of them were specifically upregulated at the transition between endodormancy and ecodormancy and downregulated in the ecodormancy (Figure 2C). Most of the predicted targets of these two miRNAs were upregulated in ecodormancy and encoded MYB TF related to phenylpropanoid pathway (Figure 3C). This is the case of MD02G1087900, MD08G1070700, MD15G1215400 and MD15G1215500, which are predicted orthologues of the proanthocyanidins biosynthesis activator of *A. thaliana* MYB123 TF [56], and MD15G1215100, MD01G1084400 and MD15G1051000, encoding putative MYB3 TF orthologues, which in *A. thaliana* represses phenylpropanoid biosynthesis gene expression [57]. Thus, the miRNAs mdm-miR858 and ath-miR858b could function as modulators of phenylpropanoid during dormancy. This modulation seems rather complex, since potential targets of mdm-miR858 and ath-miR858b act as either activators or repressors in the phenylpropanoid pathway.

Several DE-miRNA target genes were included in groups of genes enriched for GO terms related to growth and plant development (Figure 3B). A group of these genes encoding NAC (NAM, ATAF and CUC) domain transcription factors are known to play functions related to meristem development in *A. thaliana* and are post-transcriptionally regulated by miR164 [58]. In particular, we found that the apple orthologues of *CUP SHAPED COTYLEDON2* (*CUC2*) (MD11G1253500), *NAC1* (MD10G1198400) and *NAC100* (MD06G1196100, MD09G1053700, MD14G1203000 and MD17G1051600) are potential targets of mdm-miR164c. MiR164 inhibits axillary formation via downregulation of targeted *CUC* genes and it is required for normal branching in *A. thaliana* [59]. The miR164-CUC module seems conserved in other plant species and in cotton (*Gossypium hirsutum*) *gh-miR164* and *gh*-*CUC2* form a regulatory gene network with *BRANCHED 1* (*BRC1*) that is believed to control branching. *BRC1* encodes a TF that controls branching in several plant species [60,61] and it is part of a GRN controlling dormancy and budbreak in apple [5]. Therefore, the downregulation of mdm-miR164c that we observed at ecodormancy (stage 4) (Table 2 and Figure 2C) could be involved in a BRC1-mediated mechanism of budbreak control in apple. These data suggest a role of miRNAs in axillary meristem development, bud dormancy and budbreak. This notion is supported by the high abundance of the miR166 we observed in apple buds during endodormancy and ecodormancy (Table S1). In *A. thaliana*, miR166 targets genes encoding HD-ZIP III transcription factors involved in the formation of axillary meristems, root lateral meristems, and determination of lateral organ polarity required for laminar outgrowth [62]. Thus, miRNAs acting in meristem development seem to play a significant role in bud developmental switches of apple.

### 3.3. A Possible Mechanism of ABA Hyposensibilization Mediated by the Mdm-miR159a-MYB33 Module during Ecodormancy

The plant hormone ABA is a central regulator of dormancy by mechanisms involving repression of cell cycle and intercellular communication via plasmodesmata [52]. In many fruit tree and vine species such as apple, peach, pear, sweet cherry and grapevine (*Vitis vinifera*), ABA levels increase during the endodormancy phase and decline towards budbreak [12,36,41,42,53,63]. We could confirm this pattern of ABA accumulation in two consecutive years through the mRNA level quantification of the ABA-responding gene *MdABA8′H* [40] (Figure 5B). Additionally, the pattern of expression of *MdNCDE1* suggests that the observed ABA increase might be triggered by cold temperatures and/or drought typical of winter [40]. In *A. thaliana*, ABA induces the accumulation of miR159, which in turn reduces the sensitivity to this hormone by repressing the expression of *MYB33* and *MYB105*. This feedback loop is essential for ABA homeostasis in plant cells [40]. MiR159 also represses the expression of *MYB65*, which together with *MYB33*, promotes programmed cell death in endosperm and anthers [64]. Notably, we found that mdm-miR159a is upregulated from endodormancy to ecodormancy in apple buds, and that at least one of its potential post-transcriptional targets (*MdMYB33*) is downregulated at ecodormancy. In this context, we speculate that hyposensibilization to ABA by *MdMYB33* downregulation might be required to prevent ABA-mediated growth inhibition at the initiation of budbreak and flowering. This could be especially important to attenuate the effect of the rise of ABA levels in growing buds caused by punctual drought and/or cold stress during the springtime. The miR159-mediated repression of *MdMYB33* mRNA is also related to juvenile-to-adult phase transition in *A. thaliana* through the transcriptional control of miR156 and its target gene *SPL9* [65]. However, although we could find small RNAs annotated as miR156 within our sequencing data (Table S1), they were not differentially expressed during the endodormancy to ecodormancy transition, which makes their participation in this biological process unlikely.

## 4. Materials and Methods

### 4.1. Plant Material and Experimental Conditions

Apical floral dormant buds from short shoots (spurs) were collected in 2018–2019 winter from *Malus domestica* cv. “Golden Delicious” trees in an orchard located at the experimental station SudExpé in Marsillargues, south of France. All trees were the same age and their phenology appeared tightly synchronized. Four time points were chosen to cover endodormancy to ecodormancy as well as the transition in between (stages 1–4, see Results). For each stage, three biological replicates were collected, each of them being a mix of nine buds from three trees. The dormant stage of the buds was evaluated under forcing conditions (16 h/8 h, light: dark photoperiod at 22 °C) using Tabuenca’s test [32,33].

### 4.2. RNA Extraction

Total RNA was extracted from ~100 mg bud powder with ‘Spectrum Plant Total RNA Kit’ (Sigma, Darmstadt, Germany) and quantified on a NanoQuant Plate instrument (TTecan, Männedorf, Switzerland). The quality of the RNA was verified in a TapeStation 4200 (Agilent Genomics, Santa Clara, CA, USA) and in an agarose gel. Small RNAs were extracted using Lexogen’s TraPR Small RNA Isolation kit (Lexogen, Wien, Austria) [31].

### 4.3. Small RNA-Seq and Data Analysis

Small RNA libraries were prepared using the Small RNA-Seq Library Prep Kit for Illumina with TraPR (Lexogen) and sequenced on an Illumina HiSeq3000 instrument (1 × 150 bp single read) at the Max Planck Genome Centre in Cologne, Germany. Raw sRNA-seq data was analysed using Mir-Island 2.0 [66]. Reads were mapped to the apple “Golden Delicious” reference genome GDDH13 v1.1 [67]. Plant mature miRNAs from the literature were obtained by combining two miRNA databases: mirBase [68] and PNRD [69], which contain respectively 308 and 208 entries for mature apple miRNAs. Identification of miRNAs within the smal RNA sequencing data was performed as follows: the Mir-Island program first identifies already known small RNAs based on data provided by the user (miRbase and PNRD). Then, it searches for de novo miRNAs by mapping small RNA reads to loci in the genome that can form a proper RNA hairpin structure that is coherent with a miRNA-producing locus. It also provides the reads of mature miRNA and miRNA* found in the sequencing for each small RNA. An independent miRNA annotation by sequence homology with known MiRNA described in miRBase and PNRD database was also done (Table S1). Mir-Island read counts were then used for differential expression analysis. This was conducted in R 4.0.3, using two common packages for RNA-seq data analysis: EdgeR 3.32.1 [70] and DESeq2 1.30.1 [71]. Counts were normalized using the trimmed mean of M values (TMM) method in EdgeR and the median of ratios method in DESeq2. Low-count tags were discarded according to each package pipeline. In both packages, counts are fitted to Negative Binomial generalized linear models. Fold-changes are estimated and miRNAs are tested for differential expression compared to the first stage (stage 1) with a likelihood ratio test (LRT). False discovery rates (FDR) are calculated using the Benjamini-Hochberg method. Heat maps of DE-miRNAs were constructed using Morpheus (https://software.broadinstitute.org/morpheus, 31 March 2021) and default parameters. RNA secondary structure was calculated using RNAfold [72].

### 4.4. In Silico Prediction of miRNA Target Genes

MiRNA targets were predicted using the psRNAtarget web interface (2017 release) [73]. We chose a cut-off of E = 3.0 for miRNA-target sequence matching. This only allows to find potential degradation by cleavage, as translation inhibition by miRNAs usually acts when there are more mismatches between the sequence. The “Golden Delicious” reference transcriptome GDDH13 v1.1 was used [67]. Public RNA-seq data [35] were used to build expression heat maps using Morpheus (https://software.broadinstitute.org/morpheus, 31 March 2021) and default parameters.

### 4.5. Gene Ontology Analyses

The GO enrichment test was done using the Bingo tool (v3.0.3) [74] implemented in Cytoscape (v3.8.2, cytoscape.org, April 2021). The GO term enrichment in the list of 319 potential DE-miRNA target genes was assessed using the hypergeometric test. Raw *p*-values were adjusted for multiple testing using the Benjamin Hochberg method [75]. A high resolution GO map can be found in Figure S1.

### 4.6. Gene Expression Quantification

RT-qPCRs were performed on a LightCycler 480 using SYBR Green fluorescence detection (Roche, Basel, Switzerland). The parameters used for large were 50 cycles and 60 °C for annealing. PCR efficiencies were estimated using the LinRegPCR program [76]. Primers were designed using Primer3 online interface [77]. Relative quantification was calculated using the ddCT method [78]. WD40 and MDH were used as reference genes [79]. A list of all primers used in this study can be found in Table S5. All statistical analyses were performed using R stats package for R 4.0.3.

### 4.7. RLM-5′RACE

To validate the predicted miRNA-induced cleavage site, a modified RNA ligase-mediated 5′ Rapid Amplification of cDNA ends was used as described in [80]. From 5 µg of total RNA, the 5′RACE protocol was performed using the GeneRacer kit (Invitrogen Life Technologies, Waltham, MA, USA), according to the supplier’s instructions except for the first two steps (decapping and phosphorylation), which are specific of intact transcripts.

In order to maximise amplification specificity, two successive rounds of PCR amplification (nested PCR) were performed using the Phusion High-Fidelity DNA Polymerase with HF buffer (ThermoScientific), primers provided in the GeneRacer kit (GeneRacer 5′ primer and GeneRacer nested 5′ primer in the first and second round, respectively) as forward primers and gene-specific primers (MYB65-GSP and MYB65-nested-GSP) targeting MYB mRNA as reverse primers.

Primer design of the MYB65-GSP and MYB65-nested-GSP primers was performed downsteam of the predicted cleavage site using the Primer3Plus tool (https://www.bioinformatics.nl/cgi-bin/primer3plus/primer3plus.cgi; 25 September 2021) [77] with the following parameters complying with the recommendations of the GeneRacer kit: amplicon size 70–150 pb, primer length 23–28 nucleotides, primer melting temperature (Tm) 60–74 °C. The sequences of the resulting primers are shown in the Table S5.

### 4.8. Cloning, Colony PCR and Sequencing

After PCR product separation by gel electrophoresis (1.5% agarose gel in 1× Tris acetate EDTA buffer, migration 100 V/1h30) and visualization under ethidium bromide/UV light, amplification products with a size compatible with the occurrence of miRNA-directed cleavage were selected. They were purified with the DNA Clean & Concentrator kit (ZymoResearch, Irvine, CA, USA), then their blunt ends were changed into cohesive ends with dATP and Diamond Taq DNA Polymerase (Eurogentec, Seraing, Belgium), thanks to its terminal transferase activity allowing TA cloning. After another purification with the DNA Clean & Concentrator kit (ZymoResearch), cloning was performed using the TOPO-TA Cloning Kit for Sequencing (Invitrogen Life Technologies) following the manufacturer’s instructions modified as follows: bacterial transformations were performed using 2.5 µL of ligation reaction with 50 µL of One Shot chemically competent cells. Transformed bacteria (50, 100 or 150 µL) were plated on a Luria-Bertani (LB) medium supplemented with 1.5% agar and with a final concentration of 100 µg/mL ampicillin.

Several produced colonies from each target gene were analyzed by colony PCR with M13 F/R primers followed by electrophoresis on a 1.5% agarose gel. Those of the PCR products displaying a size compatible with the predicted miRNA-directed cleavage were purified using the QIAquick PCR purification kit (Qiagen, Hilden, Germany). Sanger sequencing of the purified amplification products was performed by Eurofins Genomics (Köln, Germany).

## 5. Conclusions

Our results revealed that at least 17 miRNAs change their pattern of expression during dormancy in apple buds. Since most plant miRNAs control the activity of multigene TF families, the activity of these 17 miRNAs is expected to have a significant effect on bud development. Thus, the knowledge produced here can pave the way for using miRNAs as a biotechnological tool for improving apple tree traits related to dormancy and budbreak. However, this will undoubtedly require a step of miRNA validation and functional characterization that could be done, for example, using gene editing technologies.

## Figures and Tables

**Table 1:** Samples used for the small RNA and sequencing results.

Sample	Date	Avg T (°C)	CH (h)	Phase	Library	Reads
Stage 1	7 January 2019	6.00	665	Early endo	A	22,353,097
					B	23,372,379
					C	22,980,048
Stage 2	4 February 2019	5.84	1098	Late endo	D	20,623,140
					E	23,353,672
					F	18,094,050
Stage 3	19 February 2019	7.98	1280	Early eco	G	23,560,798
					H	16,446,157
					I	20,745,045
Stage 4	4 March 2019	11.28	1395	Late eco	J	20,396,876
					K	22,812,563
					L	21,813,675

Sample: name of the sample, Date: date of sampling, Avg T: average of the day-temperature at the sampling date, CH: chilling hours at the sampling date, Phase: physiological stage of the trees based on a forcing test (endo: endodormant, eco: ecodormant), Library: name of the library for sequencing, Reads: number of reads obtained per library.

**Table 2:** Top DE-miRNAs.

			EDGER				DESEQ2	
Name (ID-Accession)	S2 vs. S1	S3 vs. S1	S4 vs. S1	*p* Value	S2 vs. S1	S3 vs. S1	S4 vs. S1	*p* Value
ath-miR858b	−0.70	1.12	−0.16	3.45 × 10^−3^	−0.56	1.17	0.01	1.04 × 10^−3^
mdm-miR159aMIMAT0025898	0.72	1.94	1.64	4.37 × 10^−3^	0.89	1.99	1.93	1.24 × 10^−3^
mdm-miR164cMIMAT0025909	−0.90	−0.22	−1.98	3.26 × 10^−3^	−0.76	−0.17	−1.77	3.71 × 10^−3^
mdm-miR171bMIMAT0025939	−0.83	0.74	−0.94	2.02 × 10^−3^	−0.70	0.78	-0.78	6.28 × 10^−4^
mdm-miR390eMIMAT0025973	−0.51	0.08	1.51	9.33 × 10^−4^	−0.44	0.16	1.70	3.99 × 10^−6^
mdm-miR482bMIMAT0026022	−0.75	0.30	−1.19	1.48 × 10^−2^	−0.63	0.33	−1.03	1.19 × 10^−2^
mdm-miR482cMIMAT0026023	−0.93	−0.04	−1.23	2.89 × 10^−2^	−0.81	−0.01	−1.07	2.47 × 10^−2^
mdm-miR5225cMIMAT0026052	−0.83	1.54	1.40	5.60 × 10^−6^	−0.70	1.56	1.60	4.76 × 10^−8^
mdm-miR7121cMIMAT0026042	−0.31	0.94	−0.59	1.17 × 10^−2^	−0.16	1.01	−0.35	4.83 × 10^−3^
mdm-miR7121eMIMAT0026044	0.30	1.59	−0.72	1.37 × 10^−3^	0.44	1.62	−0.52	1.02 × 10^−3^
mdm-miR858MIMAT0026070	0.59	2.51	−0.26	3.81 × 10^−4^	0.77	2.57	−0.05	3.12 × 10^−3^
mtr-miR4414a-5p	0.59	1.88	0.29	2.22 × 10^−3^	0.75	1.94	0.47	9.98 × 10^−4^
peu-miR2910	−0.01	1.33	0.17	2.05 × 10^−2^	0.11	1.37	0.39	6.48 × 10^−3^
ptc-miR159d	−0.11	0.70	1.92	2.53 × 10^−4^	0.02	0.77	2.16	1.78 × 10^−7^
sit-miR05-npr	0.44	−0.99	−0.54	4.77 × 10^−2^	0.56	−0.94	−0.26	2.67 × 10^−2^
t00205877_x43290	0.45	0.31	−1.00	1.33 × 10^−2^	0.59	0.38	−0.79	3.48 × 10^−3^
t08515395_x8579	0.20	−0.01	−1.67	6.17 × 10^−3^	0.31	0.03	−1.43	7.52 × 10^−3^

Name (ID-accession): ID and accession according to the miRBase nomenclature. S1: Stage 1; S2: Stage 2; S3: Stage 3; S4: Stage 4. Numeric values correspond to Log2 of the Fold Change (Log2FC) between stages.

## Data Availability

The small RNA sequencing datasets supporting the results of this article are available in NCBI’s Gene Expression Omnibus (GEO) repository though the GEO Series accession number GSE189658.

## Supplementary Materials

The following are available online at https://www.mdpi.com/article/10.3390/plants10122665/s1. Figure S1: A high resolution image of the GO enrichment map shown in Figure 3B. Table S1: List of small RNAs identified in the small RNA-seq experiment. Table S2: List of detected DE-miRNAs using EdgeR and Deseq2 programs. Table S3: List of potential miRNA target genes identified using psRNAtarget. Table S4: Results of the GO enrichment analysis done on the predicted DE-miRNA target genes. Table S5: List of primers used in this study.
