# Supplementary figures and images for: The Identification of Small RNAs Differentially Expressed in Apple Buds Reveals a Potential Role of the Mir159-MYB Regulatory Module during Dormancy
Source: Plants (Basel). 2021 Dec 3;10(12):2665. doi: 10.3390/plants10122665 (PMC8703471; doi:10.3390/plants10122665)

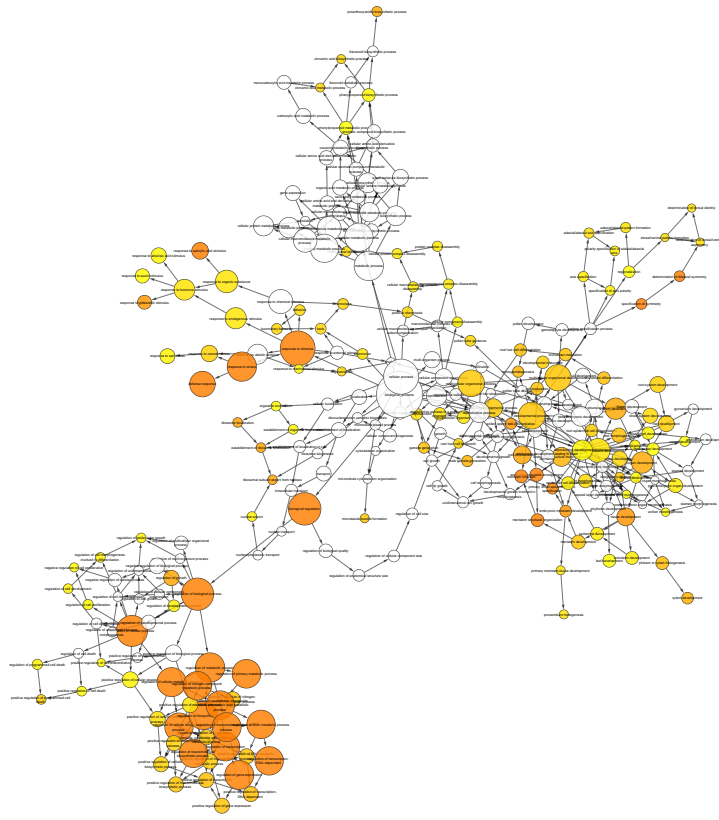

Supplement: Supplementary file 1 [file plants-10-02665-s001.zip › Sup Figure 1.pdf]
